# The Effect of Different Periodization and Modes of Concurrent Strength and Endurance Training on Double Poling Performance and Body Composition in Adolescent Cross-Country Skiers

**DOI:** 10.3390/sports10020015

**Published:** 2022-01-20

**Authors:** Eno Vahtra, Rasmus Pind, Evelin Mäestu, Priit Purge, Priit Kaasik, Jarek Mäestu

**Affiliations:** Institute of Sport Sciences and Physiotherapy, Faculty of Medicine, University of Tartu, 51008 Tartu, Estonia; eno.vahtra@ut.ee (E.V.); rasmus.pind@ut.ee (R.P.); evelin.maestu@ut.ee (E.M.); priit.purge@ut.ee (P.P.); priit.kaasik@ut.ee (P.K.)

**Keywords:** cross-country skiing, heavy strength training, body composition, maximal aerobic performance, lean mass

## Abstract

The aim of the study was to compare the effects of different types and periodization of strength training on body composition and maximal aerobic performance in 10-week training period in adolescent XC skiers. Twenty-eight adolescent competitive cross-country skiers, including 10 females (age 17.9 ± 1.8 years; body mass 69.6 ± 9.7 kg; height 1.77 ± 0.1 m; training experience 8.6 ± 3.2 years) took part in this study. Pre-and post-intervention performance was measured with the incremental exercise test (Pmax) on a double poling ski ergometer. Changes in body composition were measured with DXA. In addition to regular endurance training, experimental group one (EXP1) performed maximal and explosive strength training two times per week, experimental group two (EXP2) performed maximal and explosive strength training 1–3 times per week, and the traditional (TRAD) group performed low intensity–high volume strength training 2 times per week. Increases in arm, trunk, and overall lean mass were found in TRAD (*p* < 0.05). Increases in arm lean-mass was found in EXP1 (*p* < 0.05), while no changes in body composition occurred in EXP2 (*p* ≥ 0.05). Pmax improved significantly in all groups (*p* < 0.05). Changes in body mass, overall and arm lean mass was related to changes in absolute performance (W; *p* < 0.05), while no relationships were found between changes in body composition parameters and relative performance (W/kg; *p* ≥ 0.05). In conclusion, different periodization of strength training led to similar improvements in double poling ergometer performance, but resulted in different changes in body composition (lean mass) in adolescent cross-country skiers.

## 1. Introduction

One of the most demanding endurance sports based on metabolic stress is cross-country (XC) skiing, resulting in high anaerobic threshold and maximal aerobic power for male and female skiers [1]. In general, XC skiing involves whole-body exercise in different techniques, intensity, and duration [2]. Racing speeds in XC skiing have improved during recent decades [2], therefore, the overall ability to perform high-intensity workloads is important [3]. Consequently, the use of double poling (DP) techniques have increased the importance of upper-body power [4,5]. Previous studies have reported a strong correlation between movement-specific upper-body strength and DP performance [4,6,7]. The specific roles of elbow, shoulder, and trunk muscles have been described as playing a key role in DP techniques’ performance [5]. Häkkinen (1989) found that elite athletes’ muscle cross-sectional area and body mass were highly correlated with force production [8]. Furthermore, it has been indicated that body composition as lean mass (LM), upper-body lean mass [9], body weight, and the resulting upper body strength and power were significantly related to better skiing performance [10,11,12,13]. However, it should be taken into account that increased lean mass might have negative effects on the skier’s performance, as higher mass is transported [3,14,15]. Furthermore, there is probably an optimal distribution of LM for XC skiers, as too much muscle mass could also lead to higher anaerobic energy usage, as higher LM is associated with higher anaerobic energy turnover [15].

In addition to lean mass, fat mass is an important predictor of endurance performance in the disciplines where body mass has to be moved [3,14]. Accordingly, body fat percentage was found to have a negative impact on ski race performance [15] specially on the uphill sections in male elite adolescent cross-country skiers [10]. Similar findings can be found from road cycling, where cyclists who specialized as mountain climbers presented with height, body mass, and BMI (body mass index) that were lower than time-trial specialists [16]. To support this, it has been suggested that XC skiers should achieve a high percentage of LM and lower their fat mass [5,14].

Due to complexities between the interaction of strength gains and changes in body composition and performance, strength training as a training model has attracted interest in improving endurance athletes’ strength abilities, both in research and in practical settings [8,11,17,18]. For current scientific evidence and practical suggestions from the field, XC skiers should build up strength progressively, as the lifted loads increase week-by-week during the preparatory period from May to August [7]. Consequently, heavy strength training may be advantageous for improving endurance performance. For example, a significant (3.0 ± 1.1%) increase in upper-body lean mass resulted in a significant increase in oxygen consumption during XC skating techniques, while remaining unchanged for running in high-level junior and senior skiers [7]. Heavy strength training may also increase muscle mass [7,18] and body mass that potentially may have negative effects on some aspects of endurance performance [13], especially in disciplines where body mass is not supported. However, the combined large volumes of endurance and heavy strength training seem to reduce the hypertrophic adaptation to strength training [7,19,20,21]. Additionally, it could be suggested that due to the possible negative effect of higher body mass and the higher neuromuscular requirements of the sport, strength-training improvements should be oriented toward neural adaptions [22]. Therefore, further investigations are warranted to study the effect of combining heavy strength training with large volumes of endurance training on body composition and endurance performance in cross-country skiing.

In XC skiing, training organization has typically used relatively constant distribution of strength training with approximately two weekly strength sessions interspersed with varying amounts of low, moderate, and high-intensity training [1,3]. However, in recent years the block periodization model has been considered by athletes, coaches, and scientists [23,24,25,26]. The main idea of the block periodization model is to concentrate small number of specific sports abilities into shorter time periods [24]. Recent research in other sports indicated that block periodization of strength training may be advantageous for improving performance [25,26]. Studies with track and field and ice-hockey athletes indicate that concentrating strength training into weekly blocks may be a more efficient model to develop strength abilities and, therefore, similar models could be investigated for XC skiing [25,26]. However, to the best of our knowledge, there are also no studies where weekly variation in heavy strength training has been studied with regard to maximal aerobic performance and the respective changes in body composition in adolescent cross-country skiers, where some natural growth of tissues might still occur.

Therefore, the aim of this study was twofold: (1) to compare the effects of different types of strength training; and (2) to compare different periodizations of heavy strength training on body composition and maximal aerobic performance in adolescent XC skiers in 10-week training period. It was hypothesized that heavy strength training would be more advantageous for changes in double poling ergometer performance in adolescent skiers due to greater neuromuscular improvement and lower effect on lean mass increase.

## 2. Materials and Methods

### Subjects

Twenty-eight adolescent competitive XC skiers, including 10 female athletes (age 17.9 ± 1.8 years; body mass 69.6 ± 9.7 kg; height 1.77 ± 0.1 m; training experience 8.6 ± 3.2 years), took part in this study. The overall number of participants was 36, but eight subjects were excluded. One due to injury (not related to the experiment), four skiers had health problems, and three participants did not complete the required number of strength sessions (minimum of 90% adherence). Before pre-testing, subjects were self-selected either to experimental group one (EXP1), two (EXP2), or traditional (TRAD) group. This was done in cooperation with their personal coaches, taking into account the skiing performance level to allow similar performances at baseline between the groups. Such a method has been used before when testing high-level athletes [7,19]. The number of female athletes between the groups was 3, 4, and 3 in EXP1, EXP2, and TRAD, respectively. The participants were all among the top 10 in their respective competition classes in national youth competitions. Two athletes participated in the adult World Championships and 10 participated in the Junior World Championships during the previous winter season. Detailed written information was given to all participants before the study, and the participants gave their written informed consent before participation. Parental approval was obtained for participants younger than 18 years. The study was approved by the Research Ethics Committee of the local national university, following the standards of the Declaration of Helsinki.

## 3. Testing Procedures

This experimental study had a pre-test to post-test three-group design, where each group was treated with a different methodology. The maximal aerobic performance test (Pmax) was conducted on a double poling ski ergometer (Skierg, Concept II, Concept, Inc., Morrisville, VT, USA) before and within 2 days following the experimental period. Participants were asked to avoid strenuous training 24 h and caffeine before 2 h beforehand and to retain their normal eating habits during the day before the measurements. The Pmax test was modified based on Alsobrook and Heil’s [4] protocol. All subjects started at 40 Watts (W) and after every successive minute, the workload was increased 15 W or 20 W depending on the athletes’ performance level. The test was stopped when the subject’s power output dropped by 5 or more W below the required workload for five or more consecutive pulls or until volitionary exhaustion. The typical error of measurement in performance in the incremental ski ergometer double poling test was determined at about 1.0% in our lab.

The athletes’ body composition was measured with a Dual Energy X-ray Absorptiometry (DXA) (Hologic QDR Discovery, Hologic Inc., Bedford, MA, USA) before the experimental period and within 2 days after the experimental period. Participants were studied in the supine position wearing minimal clothes and no shoes. Participants were positioned and full-body scanned according to the operator’s manual using the standard analysis protocol. For DXA measurements, the coefficients of variations for body composition were less than 1.5%.

### 3.1. Experimental Period

During the 4 weeks before the experimental period, the subjects had regularly performed only endurance exercises and circuit-type muscular endurance training with low loads and high repetitions (15+ reps), using mainly low-speed velocities, and performed endurance exercise mainly on low intensities. The experimental protocol was designed so that the overall number of strength training sessions during the 10-week experimental period were similar between the three groups. Strength sessions in EXP1 and EXP2 consisted of one set of muscle endurance exercises using the circuit method for general warm-up with low 25+ RM (repetition maximum) loads. Maximal strength was developed with 5–6 exercises with loads of 4 RM to 10 RM, using 2–3 sets with 2–3 min rest between sets. Every repetition was executed with high intended movement velocity during the concentric phase [6,8]. Additionally, every strength training consisted of 3–4 explosive exercises with low loads, for example, including medicine ball, bodyweight, different jumping exercises, and short sprints [13]. Cross-country skiing-specific strength exercises were selected and included bar-tips, chin-ups, bench pulls, standing double poling, etc. The subjects were subjected to increase their RM loads during strength training sessions to ensure progress [27] while maintaining the number of repetitions. The difference in strength training programs between EXP1 and EXP2 groups was the number of weekly strength sessions. EXP1 group had two strength training sessions per week during the whole 10-week period, while in EXP2, the number of strength training sessions changed from 1 to 3 sessions per week (Table 1). To ensure that the athletes were in the same physical condition, before the post-test two weekly strength sessions were executed on the last week of the experiment for EXP2.

The TRAD group continued their usual muscular-endurance type of strength training twice per week, which did not include any heavy and/or explosive strength training. During the strength sessions, athletes in TRAD conducted more than 15 reps, using mostly circuit method, where 30–50 s of work alternated with 30–50 s of rest periods. Exercises were chosen similar to EXP1 and EXP 2 groups. Subjects in the TRAD group were asked to do as many repetitions as possible during their work time. Athletes were encouraged to add lifted loads after every 3 weeks to ensure progress [28].

Endurance training programs were similar between the groups in terms of training volume. Training intensity was performed in the low aerobic domain with intensities slightly below or around aerobic threshold intensities. One weekly session was performed at a constant intensity in the moderate zone for 60 min. Additionally, all three experimental groups performed short (6–10 s) sprints during their endurance training sessions.

For the calculation of the training load, athletes kept training diaries throughout the 10-week period and reported the mode and duration of each training session. Internal training load (ITL) was calculated using the session RPE method (sRPE) and answering the question: “How hard was your workout?” 30 min after the end of each training session with the respective number ranging from 0 to 10 [29] and calculated by multiplying the session rating by the duration of the training session [29].

### 3.2. Statistical Analysis

Statistical analysis was performed using IBM-SPSS Statistics for Windows version 20.0, 2012 (SPSS Inc., an IBM Company, Chicago, IL, USA). Descriptive analysis included means ± standard deviation (SD) for measured variables. Pre- and post-intervention measurements between groups were controlled for age, gender, and baseline, and within-group measurements were controlled for gender and baseline using a repeated measures ANOVA test. Descriptive analysis included means ± standard error (SE) for measured variables. Partial correlation coefficients with age and sex as covariates were used to detect the relationships between body composition changes and performance changes. A *p* < 0.05 criterion was used for establishing statistical significance. With an alpha criterion of 0.05 and power of 0.8, the power analysis indicated that a minimum of 8 subjects were needed to reach 0.8 statistical power with 3.6 and 4.0 W/kg.

## 4. Results

No differences were found in anthropometry and training experience at baseline between the studied groups (*p* ≥ 0.05). However, subjects in TRAD were slightly younger than subjects from EXP1 18.9 ± 0.6 years vs. TRAD 16.9 ± 0.6 years (*p* = 0.024). Mean body mass for all subjects was 69.6 ± 1.8 kg at baseline and was not different between the groups; *p* ≥ 0.05). There were no significant differences (*p* ≥ 0.05) in the overall weekly training volume, strength training volume, and load during the experimental period between the groups (Table 2). Additionally, there were no significant differences (*p* ≥ 0.05) between the experimental groups in overall training volume and in the mean number of weekly training (8.0 ± 2.9; 7.8 ± 2.6; 8.3 ± 2.6 for EXP1, EXP2, and TRAD, respectively) during the experimental period (*p* ≥ 0.05).

When analyzing body composition changes separately between males and females, all lean mass parameters increased significantly (*p* < 0.05) for both groups, except for leg lean mass that remained unchanged (*p* = 0.123 and *p* = 0.958 for male and female subjects, respectively). However, fat mass changes were different between the groups (*p* ≥ 0.05). Significant changes were found in males where fat mass decreased from 11.5 ± 0.5 to 11.1 ± 0.4; *p* = 0.005), while the change was not significant for females (from 13.0 ± 1.3 to 13.4 ± 1.1 kg; *p* = 0.148). Therefore, all comparisons between the experimental groups were controlled for sex and baseline values for the measured parameters.

Changes in body composition parameters between the experimental groups are presented in Table 3. Lean body mass, arm lean mass, and trunk lean mass were significantly increased in the TRAD group (*p* < 0.05). Arm lean mass was also increased in EXP1 after the 10-week training period (*p* = 0.030), and there was a tendency (*p* = 0.055) towards lean mass growth in EXP1. No changes were found in body composition values in the EXP2 group (*p* > 0.05).

**Figure 1:**
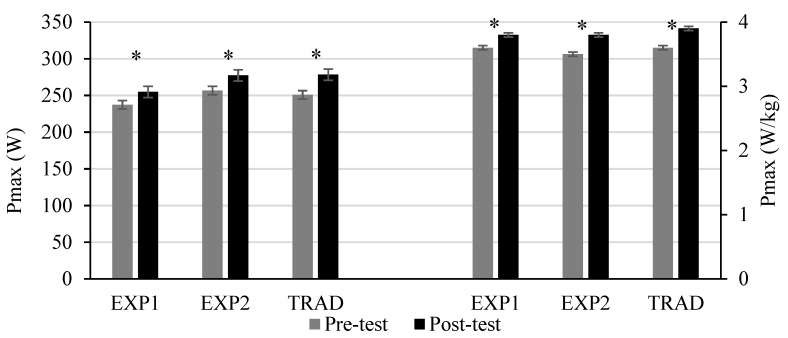
Maximal (Pmax) and relative (Pmax/kg) performance output on double poling ergometer before and after the 10 week intervention period in adolescent XC skiers. EXP1, group performing two heavy strength training sessions weekly; EXP2, group performing 1–3 heavy strength training sessions weekly; TRAD, group performing 2 weekly sessions of low-intensity high volume strength training; * significant difference between pre-test to post-test (controlled for gender and baseline value); (*p* < 0.05).

All groups improved significantly their double poling ski ergometer performance. The increase was significant for both, maximal (*p* = 0.007, *p* = 0.003, *p* = 0.000, for EXP1, for EXP2 and TRAD, respectively) and for relative performance values (*p* = 0.001, *p* = 0.000, *p* = 0.000, for EXP1, EXP2 and TRAD, respectively) (Figure 1) and the direction of the change of performance between the groups was not different.

When performing the correlation analysis between changes in body composition parameters and changes in performance, we found that changes in body mass (r = 0.432), lean mass (r = 0.618), arm lean mass (r = 0.565), and trunk lean mass (r = 0.383) were significantly related to changes in maximal performance (W) on double poling ski ergometer. However, no relationships were found between changes in body composition and changes in relative performance (W/kg).

## 5. Discussion

To the best of our knowledge, there is no study that has evaluated the effect of different strength training periodization modes on body composition and aerobic upper body performance in adolescent XC skiers during the beginning of the preparatory period. The main finding of the study was that there were no significant differences in the changes in maximal aerobic performance between the studied groups on a double poling ergometer after 10 weeks of training. However, the training period resulted in different changes in body composition, especially in the group that used strength training with low-intensity and high repetition mode. Therefore, the hypothesis that heavy strength training has a higher effect on performance compared to traditional strength training was partly rejected.

Generally, it is accepted that a higher level of performance components leads to better sporting performance. Therefore, strength training is frequently included in XC skier’s daily routine to cope with the overall changes in XC skiing towards more powerful upper bodywork. Some previous studies have found that upper body heavy strength training increases time to exhaustion (TTE) on double poling ergometer [6] and on double poling roller skiing [17]. Additionally, Carlsson et al. [30] found that heavy strength training and ski-ergometer training both improved double poling TTE in junior XC skiers. In the current study, all three groups improved their performance (*p* < 0.05), adding to the growing literature about the positive effects of strength training on aerobic performance in general, and also for XC skiers. However, it should be considered that the increase in performance test in present study could be partly explained to some degree as logical outcome, because the experiment started in the first phases of the preparatory period in late May, where the overall performance gains are somewhat easier to achieve. Similarly, the study of Losnegard et al. [7] found performance gains in double poling performance for both strength and control group during the preparatory period using mixed groups of male and female elite Norwegian skiers. Absolute performance (W) improvements were 6.9, 7.5, and 10% for EXP1, EXP2, and TRAD, respectively. In previous studies, the of improvements in time to exhaustion were higher, but due to different testing procedures makes the outcomes difficult to compare [6,17,30]. Furthermore, the time to exhaustion test is generally more sensitive to changes in performance and therefore, changes in these tests are also greater compared to consistent time or distance tests. It is also worth considering that the overall load of strength training was similar between the groups (Table 2). Therefore, the main contributor for the change in double poling performance in adolescent XC skiers might be the overall load rather than the used mode of the strength training. However, the effect on performance might also be induced by different muscle fibers or fiber recruitment. As it has previously been indicated that XC skiers with lower performances might benefit more from strength training, the performance level of the subjects should also be considered. However, as the direction of the changes in performance in the current study were similar, it could be suggested that strength training has a supportive effect on double poling performance.

It should be considered that the accompanying increases in strength abilities that also accompany increases in lean mass may have a negative impact on the economy (higher mass has to be moved) of the skier and, therefore, on overall skiing performance. Studies have indicated that changes in skiing performance may be accompanied with [7,17] or without [18] changes in body composition after the inclusion of strength training in adult XC skiers [7,17]. The extent of overall lean mass growth in the present study was comparable with previously mentioned studies in the TRAD and EXP1 groups [7,17]. However, significant changes in body composition were not found in the EXP2 group, despite similar overall load and volume of strength training. In the current study, EXP1 and EXP2 strength training programs consisted of heavy and explosive strength exercises. In both groups, the high intended movement velocity was executed for developing muscle power. However, a significant increase was found in arm lean mass in EXP1(*p* = 0.030), and a tendency (*p* = 0.055) towards lean mass growth in EXP1. At the same time no change was found in EXP2 despite the groups performed the same number of strength sessions during the 10-week period, however, the number of weekly strength training varied for EXP2 (Table 1). Those differences are difficult to explain, but it has previously been indicated that the high volume of endurance training can negatively affect strength gain and muscle hypertrophy [7,19]. It could also be argued that during the weeks with only one strength training (EXP2), the higher relative amount of endurance sessions could have had a protective effect on muscle hypertrophy, or the low number of strength sessions were not sufficient for muscle hypertrophy in general. Moreover, it has been suggested that high volume of endurance training may interfere with muscle growth while performing strength and endurance training concurrently, and a higher number of weekly strength training does not necessarily mean a higher advance in muscle hypertrophy [31].

At the same time, high volumes with low resistance exercises were used for the development of strength in the TRAD group. Generally, it is accepted that loads of ≥65% 1 RM [32] need to be used to induce muscle hypertrophy, but some recent authors have pointed out that low loads may also induce muscle growth [28,33] even for type I muscle fibers [33]. This might give an explanation for lean mass growth in TRAD (Table 3). This, however, might not fully be transferred to performance improvement because increases in work rate will recruit higher motor units, but with low load strength training, these motor units get less stimulus and might be relatively weaker [33]. It is also important to point out that no changes were found for leg lean mass in TRAD, which was the same for all study groups (*p* ≥ 0.05).

In the current study, correlation analysis showed associations between the increase in body mass, different lean mass components, and between increases in absolute double poling performance (W). Previous investigations have found similar outcomes indicating the importance of the lean mass for performance improvement [7,9,10,11]. It should be considered that when performing on a double poling ergometer, the body mass of the skier is not moved, while in XC skiing the body mass of the athlete is transported. Moreover, increases in lean mass or body mass might even have a positive impact on maximal performance on a double poling ergometer as the skier can use higher mass to support the work when pulling down the handlebars of the ergometer. This might be one explanation why the increase in performance in TRAD was comparable with the EXP1 and EXP2 groups. However, in real-life conditions, the skier has to move his/her mass during the competitions and therefore, relative performance values should be considered. However, as we did not find correlations with the changes in different lean mass parameters and changes in relative performance per body weight, we could suggest that strength training which is targeted towards neural adaptation, would be preferable in adolescent skiers compared to low intensity-high volume strength training. It is of importance that these results must be transferred from the lab to real-life conditions. Unfortunately, we were not able to test the performance in roller skies to support this suggestion.

When interpreting the results of the current study, some limitations should be considered. Firstly, 10 female and 18 male subjects participated in the study and we used mixed groups when studying training effects. Therefore, gender effect might play a role here. However, it has been found heavy strength training induced similar outcomes with female and male cyclists on body composition and performance parameters [34] utilizing a similar training program. In contrast, in cross-country skiing, it has been suggested that female skiers benefit more from strength training due to their possible lower strength levels, especially in the upper-body [6,7,12]. In our study, we found similar changes in lean mass when comparing males and females and the magnitude of the performance change was similar between the sexes after the study period. Therefore, we also used the correction for sex and baseline status when performing our analysis to minimize the possible effect of the use of mixed groups.

Additionally, the maturation state of subjects might have played a role in the outcomes of the study, as some growth might still be expected at that age. The mean age of the athletes was 17.9 years and, therefore, maturation or the role of growth was probably not a significant contributor. However, we added age as a cofactor to our statistical calculations to control for this possible effect. In the current study, no strength measurements were executed and, therefore, we don’t know how strength abilities developed during the 10-week period. Knowing the amplitude of changes in strength abilities could bring some additional insight into sports training. The strong aspect of the study was that we were able to match the training volume and training load from strength training and the overall training volume between the groups indicating that the overall work done between the groups was similar, which might help to interpret the findings.

## 6. Conclusions

The results of the study indicated that performing different modes or periodization of strength training concurrently to endurance training result in similar increases in double poling ergometer performance in adolescent XC skiers. However, different increases in lean mass occurred as a result of training. Therefore, the distinct outcome that occurred in changes in body composition might favor coaches and athletes to choose different approaches when planning the strength training for that particular age group. Based on the results of the current study, heavy strength training will result in the improvement of double poling ergometer performance without changes in lean mass. Practitioners should choose the most relevant methods to aim for the desired outcomes the sport requires and to take into account the athletes’ needs.

## Figures and Tables

**Table 1:** The weekly number of strength training sessions.

	Week 1	Week 2	Week 3	Week 4	Week 5	Week 6	Week 7	Week 8	Week 9	Week 10
EXP1	2	2	2	2	2	2	2	2	2	2
EXP2	3	1	2	3	1	2	3	1	2	2
TRAD	2	2	2	2	2	2	2	2	2	2

EXP1, group performing two heavy strength training sessions weekly; EXP2, group performing 1–3 heavy strength training sessions weekly; TRAD, group performing 2 weekly sessions of low-intensity high volume strength training.

**Table 2:** Weekly training characteristics during the 10-week intervention period for all three experimental groups (Mean ± SD).

Training Characteristic	EXP1	EXP2	TRAD
Training volume (h)	13.8 ± 1.9	13.9 ± 3.8	14.5 ± 1.3
Strength training load (AU)	887 ± 178	860 ± 178	870 ± 140
Strength training volume (h)	3.0 ± 0.3	3.1 ± 0.6	3.2 ± 0.5

EXP1, group performing two heavy strength training sessions weekly; EXP2, group performing 1–3 heavy strength training sessions weekly; TRAD, group performing 2 weekly sessions of low-intensity high volume strength training. h, hours; AU, arbitrary unit.

**Table 3:** Changes in body composition parameters and in maximal aerobic power during the 10-week period (Mean ± SE).

Body Mass Component	EXP1 (*n* = 8)		EXP2 (*n* = 10)		TRAD (*n* = 10)	
	PRE	POST	PRE	POST	PRE	POST
Body mass (kg)	66.0 ± 1.7	66.6 ± 1.8	72.5 ± 1.6	73.0 ± 1.6	69.7 ± 2.9	70.4 ± 2.9
Fat mass (kg)	11.5 ± 0.5	10.9 ± 0.4	13.6 ± 1.0	13.2 ± 0.9	11.7 ± 0.7	11.4 ± 0.7
Lean mass (kg)	50.6 ± 1.2	51.6 ± 1.4	54.7 ± 0.9	55.5 ± 1.0	**53.7 ± 2.2**	54.8 ± 2.3 *
Arm LM (kg)	**5.5 ± 0.2**	5.7 ± 0.2 *	6.3 ± 0.1	6.3 ± 0.1	**5.9 ± 0.3**	6.2 ± 0.3 *
Trunk LM (kg)	25.0 ± 0.5	25.5 ± 0.6	26.8 ± 0.6	27.5 ± 0.6	**26.8 ± 1.1**	27.4 ± 1.1 *
Leg LM (kg)	17.2 ± 0.5	17.4 ± 0.7	18.5 ± 0.3	18.7 ± 0.3	18.0 ± 0.8	18.1 ± 0.9

EXP1, group performing two heavy strength training sessions weekly; EXP2, group performing 1–3 heavy strength training sessions weekly; TRAD, group performing 2 weekly sessions of low-intensity high volume strength trainings. PRE, testing before intervention, POST, testing after intervention. * significant difference between pre-test and post-test (controlled for gender and baseline); (*p* < 0.05). Additionally, we found a tendency (*p* = 0.055) in the reduction in fat mass in EXP1.

## Data Availability

The data presented in this study are available on request from the corresponding author.
